# First Report of the Immature Stages of the Leaf-Mining Genus *Subclemensia* Kozlov, 1987 (Lepidoptera: Incurvariidae), with a Re-Illustration of the Type Species and a Generic Concept Discussion Based on Immature Characters

**DOI:** 10.3390/insects13050440

**Published:** 2022-05-06

**Authors:** Tengteng Liu, Xiaoping Geng, Ye Tang, Baozhu Li, Haixin Zhang, Kaijian Teng

**Affiliations:** 1 College of Life Sciences, Shandong Normal University, Jinan 250014, China; 17852185084@163.com; 2 College of Manager Training, Ministry of Agriculture and Rural Affairs of P.R. China, Beijing 102208, China; guanzhongtang@126.com; 3 Kuancheng Binggou Forest Farm, Chengde 067600, China; kuanlinlbz@126.com; 4 Kuancheng Forestry and Grassland Administration, Chengde 067600, China; kuanchengsenfang@163.com

**Keywords:** leaf mine, portable case, DNA barcode, genus, morphology

## Abstract

**Simple Summary:**

Caterpillars and pupae are important sources of evidence for the identification of primitive moths, and they can often provide quite different but useful morphological characters from those of adults. Incurvariidae is one of the most primitive groups of extant Lepidoptera, but half of the genera lack any information on caterpillars and pupae. It is important to increase the knowledge on the immature stages of Incurvariidae. *Subclemensia* Kozlov, 1987 is one of the monotypic genera in Incurvariidae. In this study, the caterpillar and pupa of the type species of *Subclemensia* are reported for the first time. The leaf mine, host plant and its biological characteristics are also provided. DNA barcodes were generated to help with the species delimitation. The adult male and female genitalia are re-illustrated by color photography to supplement the original line drawings.

**Abstract:**

The immature stages of primitive Lepidoptera can provide quite different but often useful morphological evidence and synapomorphies from those of adults. Incurvariidae is one of the most primitive lineages of extant Lepidoptera, which is species-poor but highly diverse, but half of the genera lack any information on immature stages. New knowledge on the immature stages of the family is expected to provide useful morphological evidence and synapomorphies to stabilize the generic nomenclature. *Subclemensia* Kozlov, 1987 is one of the monotypic genera in Incurvariidae. In this study, the immature stages of the type species of *Subclemensia* are reported for the first time. The leaf mine, host plant and its biological characteristics are also provided. DNA barcodes were generated to aid the species delimitation. The adult male and female genitalia are re-illustrated by color photography to supplement the original line drawings. The generic concepts of *Subclemensia* and other related genera are discussed based on immature characters.

## 1. Introduction

Incurvariidae is a species-poor but highly divergent family. The family has 54 species in 12 genera [1,2,3], including *Plesiozela* Karsholt & Kristensen, 2003 [4], which was recently moved into the family based on transcriptome phylogenetic analysis [5]. Most of the genera are represented by less than five species, or even by a single species. Immature stages of primitive Lepidoptera can provide quite different but often useful morphological evidence and synapomorphies from those of adults. However, little is known about the immature stages of half of the genera of Incurvariidae, such as *Plesiozela*, *Protaephagus* Scoble, 1980 [6], *Perthida* Common, 1969 [7], *Simacauda* Nielsen & Davis, 1981 [8], *Basileura* Nielsen & Davis, 1981 [8] and *Subclemensia* Kozlov, 1987 [9]. Therefore, it is of specific importance to discover the immatures of such a divergent family and to test the morphological synapomorphies to stabilize the generic nomenclature.

*Subclemensia* Kozlov, 1987 was established as a monotypic genus, of which *S. taigae* Kozlov, 1987 is the type species [9]. *Subclemensia* is closely related to *Paraclemensia* Busck, 1904 by the male genitalia and the absence of protibial epiphysis, and to *Alloclemensia* Nielsen, 1981 by the forewing markings. *Subclemensia* combines features of the two genera indicated and occupies an intermediate position between them [9]. Further evidence is necessary to stabilize the status of *Subclemensia*. Nielsen (1981) revised the genus *Alloclemensia* and proposed eight autapomorphies, three of which: (1) the prominent caudal process from the middle of the posterior margin of the tegumen, (2) the reduced juxta and (3) the shorter apophyses anteriores than apophyses posteriores, are most remarkable. Later, he revised *Paraclemensia* and proposed four autapomorphies: (1) a combination of the absence of whitish markings on the forewing and metallic luster; (2) protibial epiphysis absence; (3) male with prominent spines on the valva arranged in distinct clusters on humps on the ventral margin of the valva; (4) medial part of the ovipositor strongly extended and almost arrowlike [10]. However, no characters of the immature stages are used to stabilize the generic concepts of the abovementioned genera.

Knowledge on the immature stages of *Subclemensia* and related genera is relatively poor compared to that of the adults. Ross (1958) described the immature stages of *P. acerifoliella* (Fitch, 1956) [11], and subsequently Pohl et al. (2015) expanded its host association and distribution [12]. Lundblad (1927, 1930) provided detailed information on the immature stages of *A. mesospilella* (Herrich-Schäffer, 1854) [13,14], and Lepiforum has also provided excellent photos (https://lepiforum.org (accessed on 15 March 2022)). Nielsen (1981) noted the biology of the immature stages of *A. unifasciata* (Nielsen 1981) [15]. No information on the immature stages of *Subclemensia* is currently available.

Recently, *S. taigae* Kozlov, 1987, the type species of *Subclemensia*, was discovered in China for the first time. The larva of this species is a serious pest to birch (Betulaceae). The present paper reports the immature stages of *S. taigae* and illustrates the portable case, the host plant and its biological characteristics. The adult male and female genitalia are re-illustrated by color photography. The generic concepts of *Subclemensia* and other related genera are discussed based on the immature characters.

## 2. Materials and Methods

More than 20 leaf mines and early-instar larvae were collected on *Betula platyphylla* in Binggou Forest Farm (118.86° E, 40.56° N), Chengde, Hebei Province, China, in May 2020, to study the characteristics of the larvae and leaf mines. In early September, the larvae in portable cases descended the tree onto the ground to find a place for overwintering. These larvae were collected and placed in an outdoor net cage, and they emerged in mid-May of the following year. Immature larval specimens were kept for dissection and morphological study. Three first-instar larvae in cases and two mature larvae were examined. All the specimens were kept at Shandong Normal University (SDNU).

Genitalia and larvae preparations were made based on the method described by Li (2002) [16], using 10% NaOH solution to boil and digest internal tissues, and then transferring to water to clean up, stain with eosin and/or thiazole black and dehydrate with anhydrous ethanol. Then, the tissue was fixed with xylene and sealed with neutral gum to create permanent slides. Venation slides were created in a similar way but could be treated without 10% NaOH. Photos of the adult specimens were taken using a Canon EOS 5D Mark IV camera plus a Canon MP-E 65 mm lens (Canon Inc., Tokyo, Japan) and a Leica M165C stereo microscope (Leica Microsystems Ltd., Heerbrugg, Switzerland) to reveal the metallic luster under different light on the wings. Larvae, portable cases, pupae and early leaf mines were photographed with a Leica M165C stereo microscope (Leica Microsystems Ltd., Heerbrugg, Switzerland). Photographs of the host plant, damage and portable cases on the ground were taken using a Canon EOS 5D Mark IV camera plus a Canon micro 100 mm lens (Canon Inc., Tokyo, Japan). Photographs of genitalia and fine structures of larvae were taken with a Leica DM750 microscope (Leica Microsystems Ltd., Heerbrugg, Switzerland). All the photographs were typeset with Photoshop^®^ CS4 software (Adobe Systems, San Jose, CA, USA). 

The genome of three larval specimens of the new species was extracted using Qiagen DNeasy Blood & Tissue Kit (Qiagen, Shildon, Germany), and the mitochondrial COI gene fragment was amplified using the primer pair HCO2198/LCO1490 [17]. The PCR system and the thermal cycling conditions followed deWaard et al. (2008) [18]. The PCR products obtained by amplification were sent to Qingke Biological Company, China, for sequencing. Thirty-one public barcode sequences, three of *S. taigae* and two of Adelidae as outgroups, were included in the analysis. All the sequences were deposited in a public dataset, DS-INCUR, in BOLD (Table 1). The alignment of the new sequences and public ones and the genetic distance analysis were performed in MEGA X [19]. A maximum likelihood tree was created using MEGA X, and bootstrap values were calculated with 1000 replicates.

Host plant family names were based on APG IV (2016) [20], and species names were based on The Plant List (2013) [21]. The morphological terminology of the adult moth was in accordance with Nielsen (1982) [10], and that of the immature stages followed Zimmerman (1978) [11,22].

## 3. Results

***Subclemensia* Kozlov, 1987**

*Subclemensia* Kozlov, 1987, *In: Cheshuekrylye Dal’nego Vostoka SSSR*: 14. Type species: *Subclemensia taigae* Kozlov, 1987, by monotypy. 

***Subclemensia taigae* Kozlov, 1987**

**Diagnosis:***Subclemensia* is monotypic, so the species is diagnosed with species from related genera. *Subclemensia taigae* has white markings on its forewings, distinguishing it from all other species of the genus *Paraclemensia*. The male genitalia of this species are similar to those of *P. oligospina* Nielsen, 1982, a species known only by the male, but can be distinguished by the following features. In *S. taigae*, the posterior margin of the tegumen is nearly straight, the distal 2/3 of the phallus has a long ridge on the outer wall and a small triangular protrusion is present near the distal end, while in *P. oligospina*, the posterior margin of the tegumen has a markedly U-shaped indentation, and there is a long and curved spine protruding from the end of the phallus, without other protrusions (Nielsen 1982: Figures 22 and 24) [10]. The ovipositor of the female genitalia of *S. taigae* is most related to that of *P. cyanea* Nielsen, 1982, a species known only by the female (Nielsen 1982: Figure 37) [10], but can be distinguished from the latter by the following features. In *S. taigae*, the vestibulum has a pair of triangular sclerotized zones, and the ductus bursae and the corpus bursae are nearly equal in width, while in *P. cyanea*, the vestibulum is completely membranous, and the corpus bursae is significantly wider than the ductus bursae (Nielsen 1982: Figure 43) [10].

The forewing markings of *S. taigae* are similar to those of *Incurvaria praelatella* (Denis & Schiffermüller, 1775) [23] but can be distinguished by the following features. The new species has dark brown cilia around the apex of the forewings and is absent of a long spine process at the end of the phallus; in *I. praelatella*, the cilia at the apex of the forewings are white, and a long spine process is present at the end of the phallus (Kuprijanov 1994: Figures 4A and 8A) [24]. In addition, the forewing markings of *S. taigae* are similar to those of *Alloclemensia unifasciata* Nielsen, 1981 [15] and *Phylloporia bistrigella* (Haworth, 1828) [25], but there are significant differences in the male genitalia (Okamoto and Hirowatari 2004: Figures 6 and 8) [26].

**Material examined.** 6♂, 1♀, CHINA: Binggou Forest Farm, Kuancheng, Chengde City, Hebei Province, 118.86° E, 40.56° N, 850–1400 m, 2020.ix, leaf mine and larva on *Betula platyphylla*, collector G. Tang, registration SDNU.Ent023842 (slide no. LIU0288♂), SDNU.Ent023843-5, SDNU.Ent023846 (LIU0282♂), SDNU.Ent023847 (LIU0287♂W), SDNU.Ent023848 (LIU0283♀). 

**Other materials.** Eight larvae, mounted in slide, 2021.viii.14, ix.9, field no. LTT01116, LTT01117, LTT01119, other information same as adult. 

**Figure 1:**
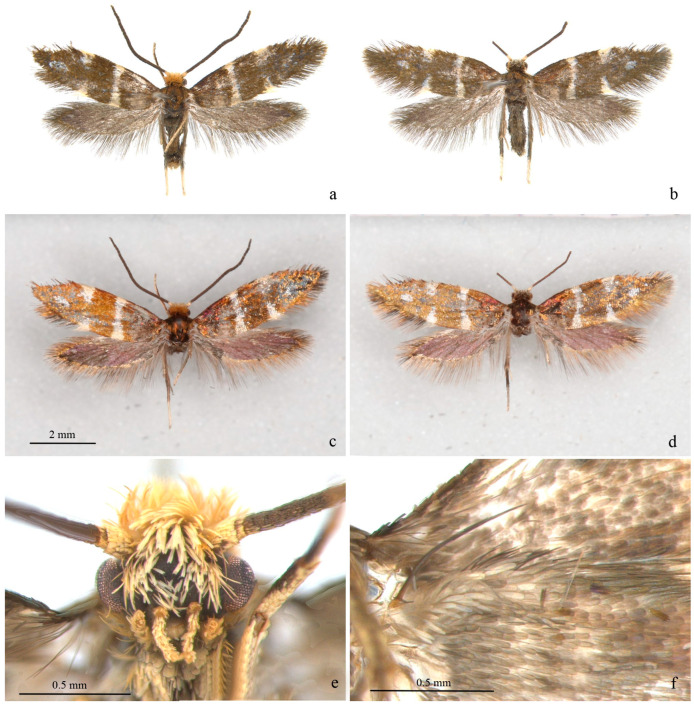
Adult of *Subclemensia taigae*. (**a**) Male, SDNU.Ent023846, photo taken under Leica M165C stereo microscope; (**b**) female, SDNU.Ent023848, photo taken under Leica M165C stereo microscope; (**c**) same specimen as (**a**), but taken under Canon EOS plus MP-E 65 mm lens to show metallic luster; (**d**) same specimen as (**b**), but taken under Canon EOS plus MP-E 65 mm lens to show metallic luster; (**e**) head, showing hair tuft on vertex and frons, maxillary palpus and labial palpus; (**f**) frenulum of male.

**Figure 2:**
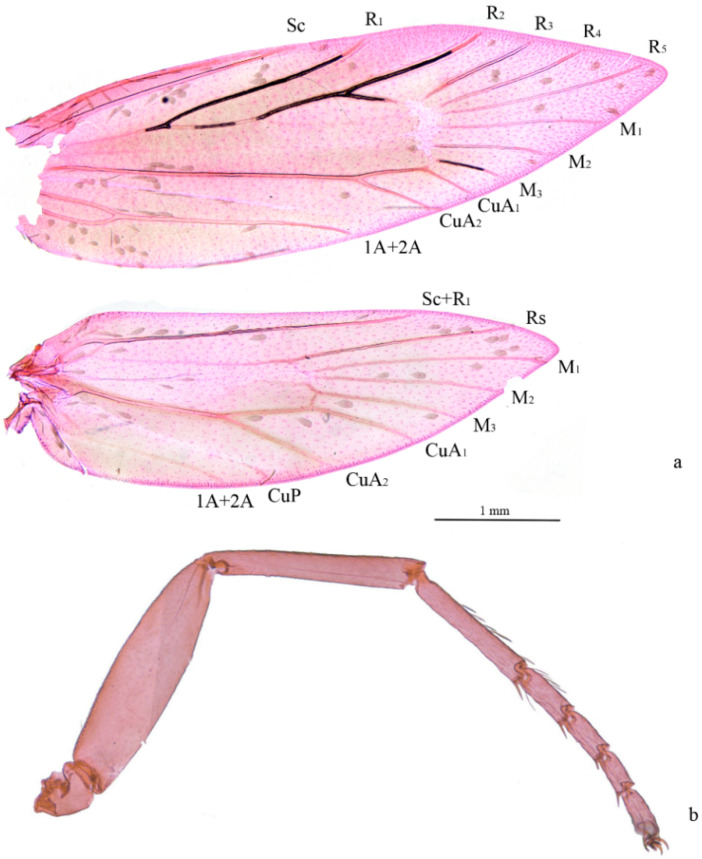
Venation and foreleg of *Subclemensia taigae*. (**a**) Venation, male, SDNU.Ent023847; (**b**) foreleg without protibial epiphysis, male, SDNU.Ent023846.

**Adult** (Figure 1 and Figure 2). Wingspan 8–9 mm. Vertex orange yellow, frons yellowish white (Figure 1e). Maxillary palpus 5 segments, pale yellow. Labial palpus 3 segments, half-length of maxillary palpus, pale yellow, tinged white distally. Proboscis reduced. Antennae 3/4 length of forewing, scape and pedicel yellowish white with golden luster, flagellum brown. Thorax and tegula brown with golden luster. Forewing blackish brown with golden luster at base, with bluish-purple metallic luster towards costa (Figure 1c,d); a transverse white fascia at basal 1/3, narrower at middle, tinged light yellow on costa and dorsum; two triangular white spots on costal 2/3 and on dorsum near tornus, tinged light yellow on wing margin, costal one slightly larger; a small white spot far before apex; cilia blackish brown, with golden luster. Hind wing gray, with bluish-purple metallic luster in disc and golden luster along margin; cilia gray, with golden luster, more prominent at base. Venation matches ground plan of the genus (Figure 2a). Leg black dorsally, white ventrally, hind tarsus white; without protibial epiphysis (Figure 2b). Abdomen dark brown dorsally, white ventrally. 

**Figure 3:**
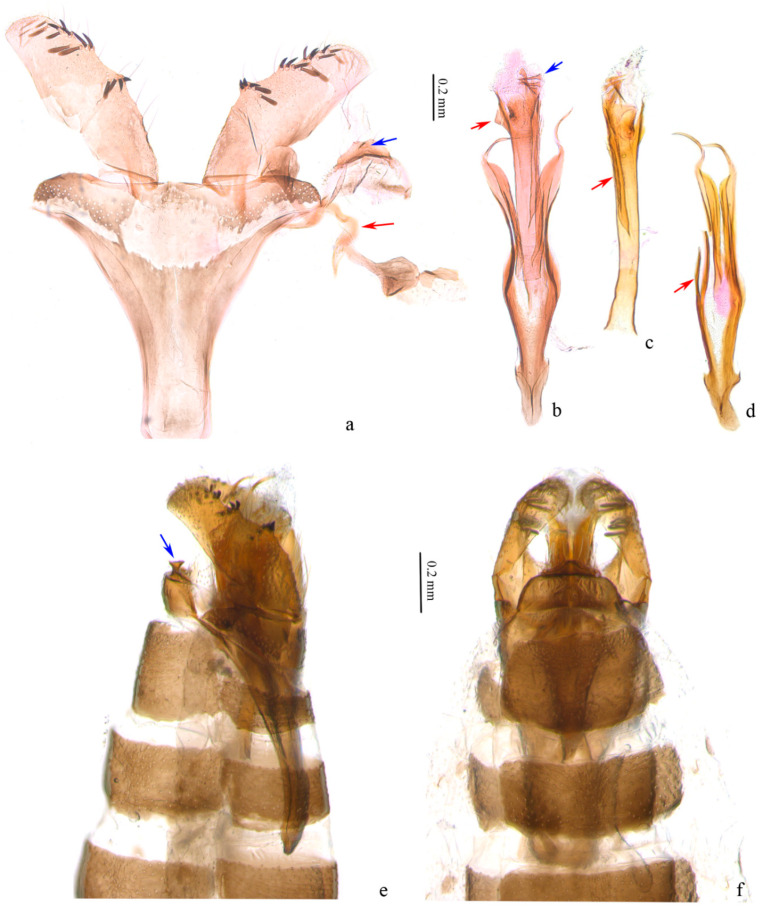
Male genitalia of *Subclemensia taigae*. (**a**) Genitalia with phallus detached, LIU0282, SDNU.Ent023846, blue arrow indicating the small sharp protrusion on tegumen, red arrow indicating the transtilla; (**b**) phallus, LIU0282, SDNU.Ent023846, red arrow indicating the small triangular protrusion, blue arrow indicating the cornuti; (**c**) phallus, LIU0288, SDNU.Ent023842, red arrow indicating the transtilla long ridge on outer wall; (**d**) juxta, LIU0288, SDNU.Ent023842, red arrow indicating the secondary branch; (**e**) genitalia in situ, lateral view, LIU0288, SDNU.Ent023842, blue arrow indicating the small sharp protrusion on tegumen; (**f**) foreleg, genitalia in situ, dorsal view, LIU0288, SDNU.Ent023842.

**Male genitalia** (Figure 3). Tegumen trapezoidal to semicircular (Figure 3a); a trapezoidal sclerotized zone on posterior margin of tegumen, with a long bristle laterally, a pair of small protrusions at middle each with a long bristle, a small sharp protrusion at middle ventrally (obvious on lateral view (Figure 3b,e)). Valva slightly rectangular, shorter than length of vinculum + saccus, widest nearly same as end of saccus; a small hump in middle and distal 1/3 of ventral margin, with 4 to 5 scale-like setae on each, an inconspicuous hump before distal end with some 10 scale-like setae around, narrower towards apex. Transtillae inverted U-shaped, in connection to base of valva by a hammer-like sclerotized band (Figure 3a). Vinculum + saccus nearly triangular (unrolling), anterior margin truncated. Phallus longer than valva, tubular, truncated at base, nearly equal width in middle, slightly wider distally; distal 2/3 with a long ridge on outer wall (Figure 3c), a small triangular protrusion at proximal end (Figure 3b), cornuti 7–8 sharp spines and numerous microspines towards base (Figure 3b). Juxta with a pair of sclerotized bands strongly narrowed and spiral on distal part, pointed distally; branches from middle of preceding bands joined to an arrow, with secondary branches from middle (Figure 3d).

**Figure 4:**
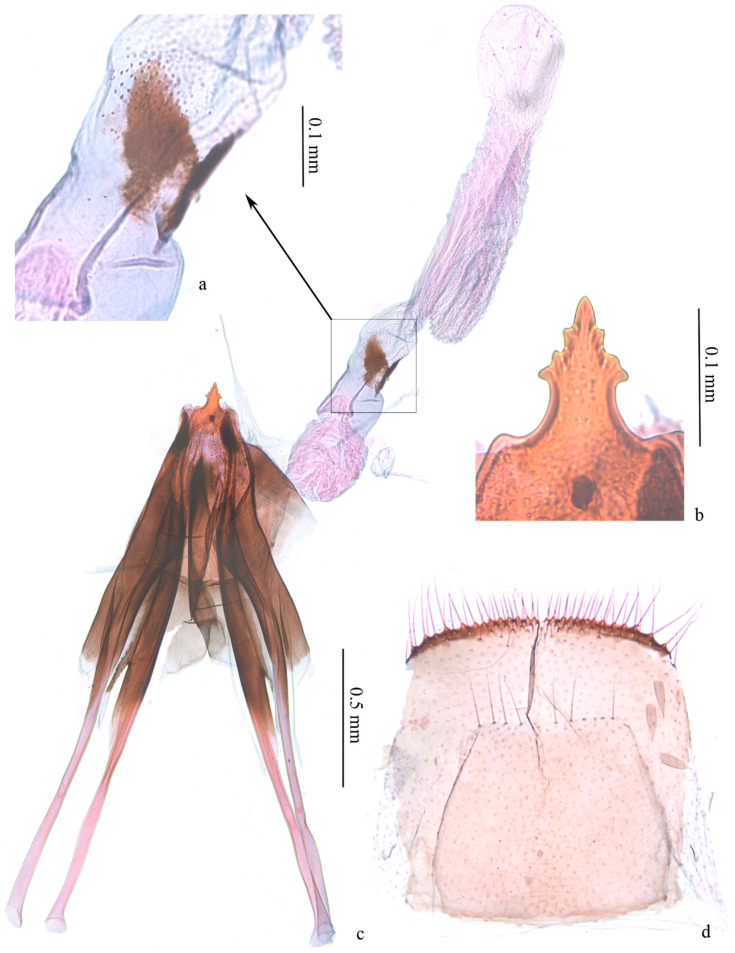
Female genitalia of *Subclemensia taigae*. (**a**) Triangular sclerotized zones on vestibulum, LIU0283, SDNU.Ent023848; (**b**) ovipositor, same slide as (**a**); (**c**) whole view of female genitalia, same slide as (**a**); (**d**) sternite and tergite of eighth abdominal segment, same slide as (**a**).

**Female genitalia** (Figure 4). Ovipositor arrow-shaped, apex blunt, with 3 serrations on each side (Figure 4b). Apophyses equal in length, with short bifurcation at middle of posterior apophyses (Figure 4c). Vestibulum nearly spherical on basal 1/3, with folds; distal 2/3 of equal width, with a pair of triangular sclerotized zones (Figure 4a), densely covered with triangular microspines nearby. Ductus bursae with dense longitudinal folds and series of microspines. Corpus bursae membranous, small, oval. Sternite and tergite of eighth abdominal segment with posterior margin almost straight (Figure 4d). 

**Figure 5:**
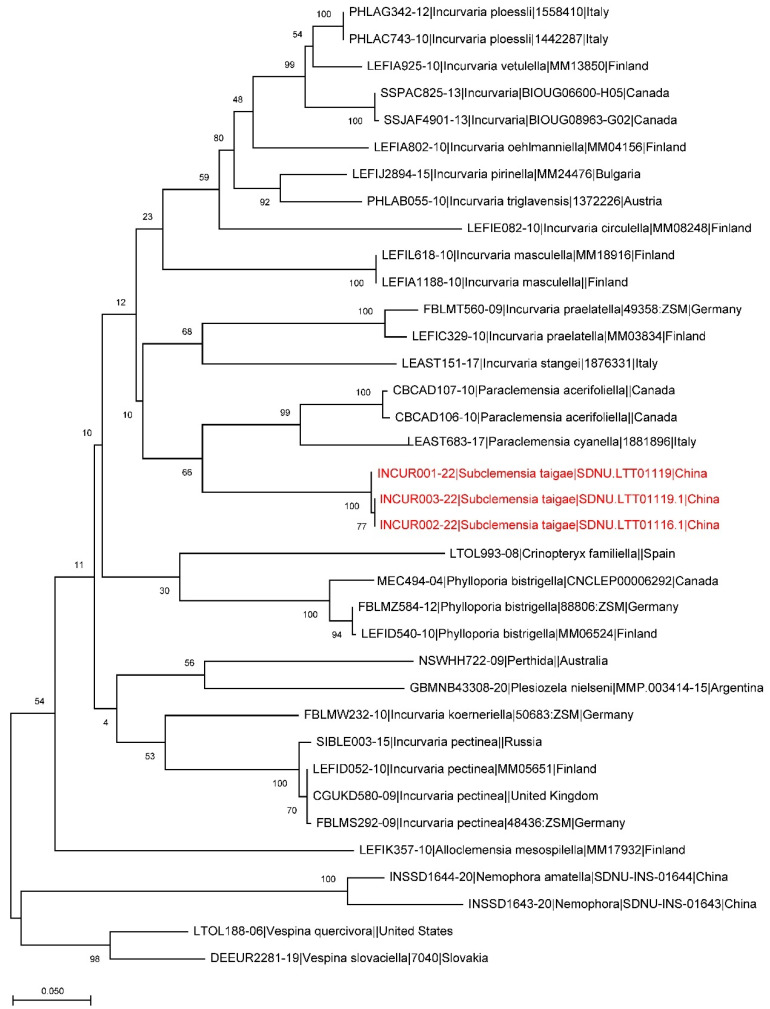
A maximum likelihood estimation based on available barcode sequences of Incurvariidae. The branches of *Subclemensia taigae* are marked red.

**DNA Barcodes**. Three barcodes were generated from larval specimens, process IDs: INCUR001-22, INCUR002-22, INCUR003-22 (Table 1). The genetic analysis also supports the separation of the new species from other related species (Figure 5). 

**Figure 6:**
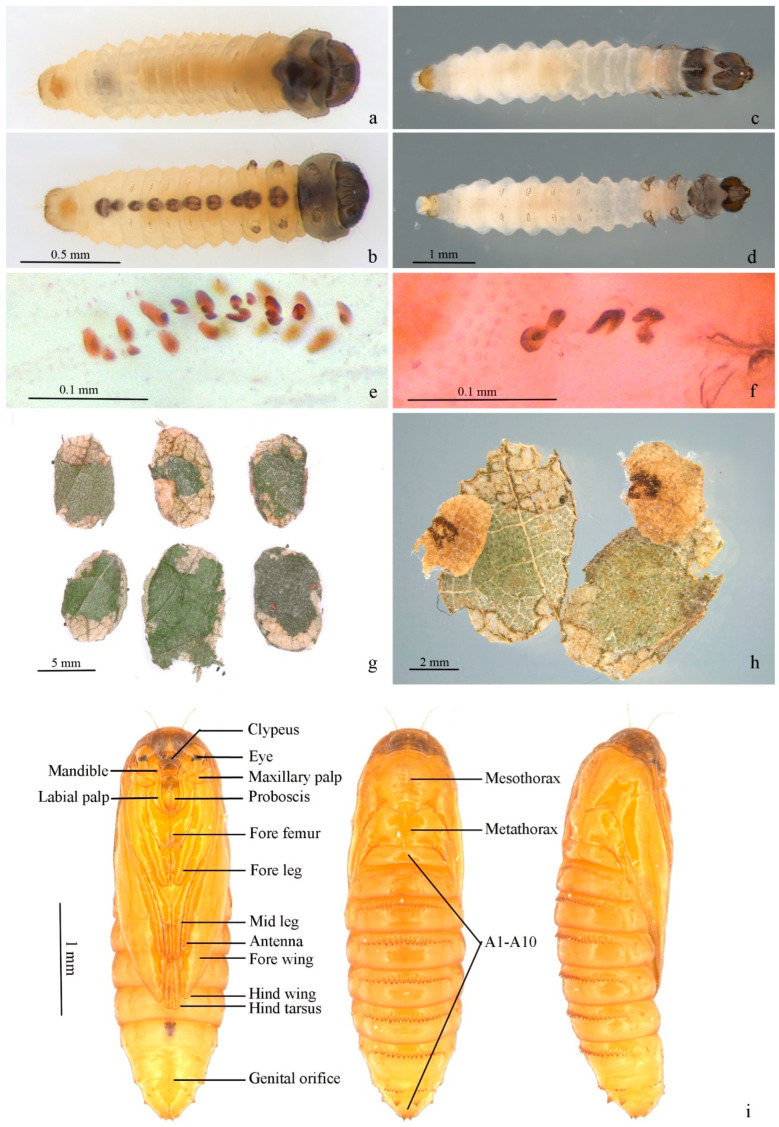
Immature stages of *Subclemensia taigae*. (**a**,**b**) First instar in case: (**a**) dorsal view, (**b**) ventral view; (**c**,**d**) mature larva, boiled in water: (**c**) dorsal view, (**d**) ventral view; (**e**) crochets on A3–6; (**f**) anal crochets; (**g**) portable case for overwintering; (**h**) opened case, showing the two layers and the feces attached on the outer surface of the inner layer; (**i**) pupa from ventral, dorsal and lateral views.

**First instar in case** (Figure 6a,b). Body length 2.20–2.30 mm, width 0.30–0.50 mm. Head capsule blackish brown, strongly sclerotized, equal in length and width, 0.30–0.33 mm. Submentum membranous, with two setae in center. Spinneret about twice as long as width. Labial palpus composed of 3 segments, stipes membranous with two setae. Mandible with 4 pairs of teeth. Body yellowish white to dark yellow. Cervical shield pronotum light brown, heavily sclerotized. Thoracic leg normal, 3 pairs, sclerotized except on basal segment, claw (pretarsus) hooked; a dark brown spot each on segment from mesothorax to A8 along ventral meson line, larger on mesothorax, metathorax and A8. Prolegs reduced, crochets pincer-like, A3–6 uniordinal, double transverse bands, anal prolegs uniordinal, single transverse band. Spiracle circular, edge black. Anal plate semi-elliptical. 

**Mature larva** (Figure 6c–f). White with dark head and cervical shield. Body length 5.67–6.00 mm, width 0.87–1.17mm. Head capsule 0.60–0.65 mm in length, 0.75–0.80 mm in width. Dark spot from mesothorax to A8 along ventral meson line equal in size. Prolegs and crochets similar to early instars (Figure 6e,f). 

**Portable case** (Figure 6g,h). Flat oval (Figure 6g). Single layer when first appears, double layers in mature stage. Inner layer smaller than outer, pale yellow, attached to outer layer by thin white mesh; larvae between inner layers. Outer layer composed of two pieces of leaf tissue in different sizes. Larvae feed in single-layered case in early stage, and some feces can be attached to outer surface of single-layered case. Outer layers can be formed by using leaf tissues in later instar; meanwhile, feces attached on outer surface of single-layered case can be covered by outer layer (Figure 6h). 

**Pupa** (Figure 6i). Body length 4.1–4.2 mm, width 1.1–1.2 mm. A7–10 fused. Light brown. Frons plump. Labrum trapezoid. Labial palpus and proboscis subequal. Maxillary palpus transverse. Antenna from dorsal side of eye to ventro-posterior edge of A3. Foreleg and midleg originated from posterior edge of eye, extending obliquely to middle ventrally, reaching posterior edge of A1 and A4, respectively; hind leg extending to middle of A5. Forewing obliquely to middle ventrally with costa along antenna, covering most of lateral side of body, extending to 1/3 of A5. Hindwing beneath forewing with only dorsum visible. Smooth dorsally, pronotum and mesonotum not clearly delimitated, mesonotum approximate pentagon. A transverse band of 16–24 teeth on anterior 1/3 of A2–8, less obvious on A2; tooth fewer on A8 and sometimes divided into 2 parts by meson line; A9 with two larger triangular teeth. Cremaster two small teeth at end dorsally.

**Distribution**. China (Hebei), Russia (Southern Primorye) (Kozlov 1987). 

**Host plant.***Betula platyphylla* Sukaczev (Betulaceae), new record. Similar damages were also found on *Betula dahurica* Pall. at the type locality in the same period. 

**Figure 7:**
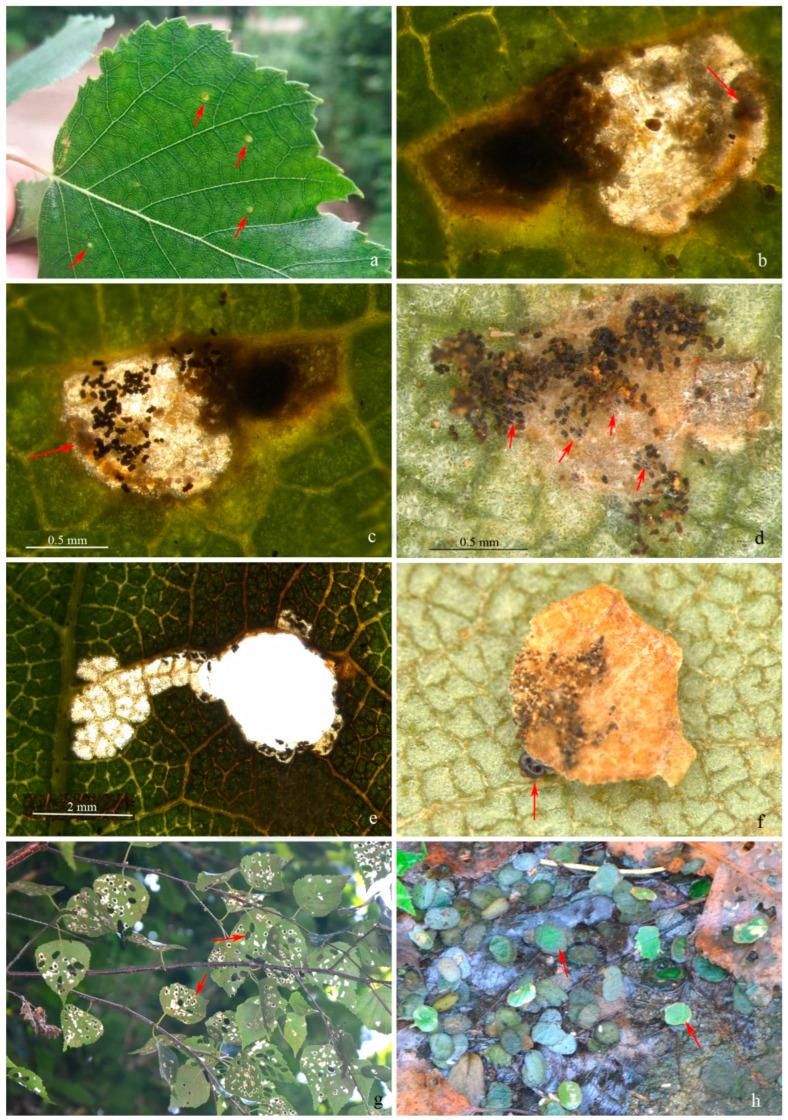
Biology of *Subclemensia taigae*. (**a**) Leaf mines of first-instar larvae as indicated by red arrows; (**b**) close-up view of early mine from upper side of leaf, arrow indicating head of larva; (**c**) close-up view of early mine from lower side of leaf, arrow indicating head of larva; (**d**) fecal pellets attached on lower surface of the mine with silk threads, arrows indicating the silk threads; (**e**) a hole on the leaf created by first-instar larva in case; (**f**) a single-layered portable case, arrow indicating the head of the larva; (**g**) damage by the larvae, arrows indicating the holes on leaves; (**h**) portable cases on the ground searching for overwintering place, arrows indicating two of the massive cases.

**Biology.** Larvae live as leaf miners in the early stage (Figure 7a–c) and later construct a portable case from the wall of the mine and feed as leaf skeletonizers (Figure 7f). Early mines are yellowish-green spots (Figure 7a) and gradually become translucent over feeding (Figure 7b,c). Larvae can expel some fecal pellets out of the mine and attach them on the lower surface of the mine with silk threads (Figure 7c,d). The mines are expanded into irregular patches until forming single-layered cases forming oval holes on the leaves (Figure 7e).

This species has one generation per year and overwinters as prepupae in a case. Emergence occurs in mid-May of the second year, and the earliest mine occurs in late May. The larvae grow very slowly during the leaf-mining stage. A significant increase in the size of the mine does not occur until mid-August. Not long after that, the larvae will make single-layered cases. In early September, the mature larvae make an outer case and fall onto the ground to find a place for overwintering (Figure 7g,h). 

## 4. Discussion

Immature stages are poorly known in Incurvariidae, with half of the genera lacking any information on immature stages. Some immature morphological features are of taxonomic value [11]. Therefore, it is particularly useful to describe the immature stages of Incurvariidae for the identification of the immature stages. Some specific characters may also be useful for testing morphological synapomorphies and thus for stabilizing the generic nomenclature. The discovery of the immature stages of *S. taigae* enables us to compare the immature characters among species from closely related genera (Table 2). 

The cut-off of a leaf is one of the synapomorphies of Incurvariidae, but the initial mine by first-instar larvae differs among genera based on available data. The initial mine is a very short and irregular corridor in *S. taigae* and never forms a slender linear mine, which is quite different from the slender initial mine of *A. mesospilella* but somewhat similar to that of *P. acerifoliella*. The features on a leaf mine are of potential value for stabilizing the generic concept. A slender initial mine is extremely characteristic of *A. mesospilella* compared with other species of related genera, and it could be a generic character for *Alloclemensia*, but this cannot be confirmed further until at least the leaf mine of a second *Alloclemensia* species is known. 

The sclerotized tergite of the prothorax is one of the larval synapomorphies of Incurvariidae; however, the degree of sclerotization on T2 and T3 varies among genera. In the type genus *Incurvaria*, the tergite of each thorax is usually sclerotized as well as the tergite of some anterior abdominal segments. Large areas of sclerotization are present on T2 and T3 in *P. acerifoliella*, but only a small sclerotized patch on each lateral side of T2 and T3 in *A. mesospilella* and *A. unifasciata* is found. No sclerotized zones are found on T2 and T3 in *S. taigae*. The status of the sclerotization on T2 and T3 seems discontinuous enough to separate *Paraclemensia*, *Alloclemensia* and *Subclemensia*. The absence of sclerotization on the tergite of T2 and T3 is probably a generic character for *Subclemensia*, while the small sclerotized patch on each lateral side of T2 and T3 is shared by species of *Alloclemensia*. However, more information on the immature stages of other species is necessary to test the hypothesis; then, the respective status of the sclerotization on T2 and T3 could be added to the generic concepts of relevant genera. 

The portable cases of mature larvae are similar among genera, but the number of the leaf pieces comprising the outer layer varies between and within genera, and sometimes within an identical species, i.e., *P. acerifoliella* (Pohl et al., 2015) [12]. Therefore, it is still early to define a generic character using portable cases based on available data. 

Pupae of *Subclemensia*, *Alloclemensia* and *Paraclemensia* share vestiges of mandibles, prominent maxillary and labial palpi, a single row of spines on A3–8 and two larger spines on A9, with the length of the antenna differing in males and females. It is difficult to trace a unique and reliable character for *Subclemensia* in pupae. Therefore, no reliable pupal character can be considered as a generic character according to current knowledge. 

## Figures and Tables

**Table 1:** Specimens and DNA barcode sequences used in the molecular analysis.

Species Name	BOLD Process ID	Collecting Date	Country	Depository
*Alloclemensia mesospilella*	LEFIK357-10	2 June 2009	Finland	University of Oulu, Zoological Museum
*Crinopteryx familiella*	LTOL993-08	3 January 2001	Spain	University of Maryland
*Incurvaria circulella*	LEFIE082-10	-	Finland	University of Oulu, Zoological Museum
*Incurvaria koerneriella*	FBLMW232-10	30 April 2005	Germany	Research Collection of Peter Lichtmannecker
*Incurvaria masculella*	LEFIL618-10	-	Finland	University of Oulu, Zoological Museum
*Incurvaria masculella*	LEFIA1188-10	19 May 2007	Finland	University of Oulu
*Incurvaria oehlmanniella*	LEFIA802-10	-	Finland	University of Oulu, Zoological Museum
*Incurvaria pectinea*	CGUKD580-09	9 February 2007	United Kingdom	Natural History Museum, London
*Incurvaria pectinea*	FBLMS292-09	15 April 2009	Germany	Research Collection of Theo Gruenewald
*Incurvaria pectinea*	SIBLE003-15	18 June 2010	Russia	Institut National de la Recherche Agronomique, Zoologie Forestiere
*Incurvaria pectinea*	LEFID052-10	16 May 2007	Finland	University of Oulu, Zoological Museum
*Incurvaria pirinella*	LEFIJ2894-15	3 May 2013	Bulgaria	Research Collection of Jari Junnilainen
*Incurvaria ploessli*	PHLAG342-12	21 July 1991	Italy	Tiroler Landesmuseen
*Incurvaria ploessli*	PHLAC743-10	21 July 1991	Italy	Tiroler Landesmuseen
*Incurvaria praelatella*	FBLMT560-09	24 June 2004	Germany	Research Collection of Alfred Haslberger
*Incurvaria praelatella*	LEFIC329-10	19 June 2006	Finland	University of Oulu, Zoological Museum
*Incurvaria* sp.	SSPAC825-13	14 July 2012	Canada	Centre for Biodiversity Genomics
*Incurvaria* sp.	SSJAF4901-13	21 July 2012	Canada	Centre for Biodiversity Genomics
*Incurvaria stangei*	LEAST151-17	23 June 2017	Italy	Tiroler Landesmuseen
*Incurvaria triglavensis*	PHLAB055-10	1 July 2009	Austria	Tiroler Landesmuseen
*Incurvaria vetulella*	LEFIA925-10	-	Finland	University of Oulu, Zoological Museum
*Nemophora amatella*	INSSD1644-20	12 May 2019	China	Shandong Normal University
*Nemophora* sp.	INSSD1643-20	1 July 2017	China	Shandong Normal University
*Paraclemensia acerifoliella*	CBCAD107-10	9 June 2009	Canada	Centre for Biodiversity Genomics
*Paraclemensia acerifoliella*	CBCAD106-10	9 June 2009	Canada	Centre for Biodiversity Genomics
*Subclemensia taigae*	INCUR003-22	14 August 2021	China	Shandong Normal University
*Subclemensia taigae*	INCUR002-22	14 August 2021	China	Shandong Normal University
*Subclemensia taigae*	INCUR001-22	9 September 2021	China	Shandong Normal University
*Paraclemensia cyanella*	LEAST683-17	20 May 2004	Italy	Tiroler Landesmuseen
*Perthida* sp.	NSWHH722-09	28 December 2008	Australia	Centre for Biodiversity Genomics
*Phylloporia bistrigella*	MEC494-04	19 June 2004	Canada	Canadian National Collection of Insects, Arachnids and Nematodes
*Phylloporia bistrigella*	FBLMZ584-12	16 July 2010	Germany	Research Collection of Peter Lichtmannecker
*Phylloporia bistrigella*	LEFID540-10	3 July 2007	Finland	University of Oulu, Zoological Museum
*Plesiozela nielseni*	GBMNB43308-20	-	Argentina	Mined from GenBank, NCBI
*Vespina quercivora*	LTOL188-06	4 December 1992	United States	University of Maryland
*Vespina slovaciella*	DEEUR2281-19	18 April 2015	Slovakia	Research Collection of Zdenko Tokar

**Table 2:** Diagnosis of immature stages of species in related genera.

	Leaf Mine before Hole of Cut-Off	Portable Case of Mature Larva	Mature Larva	Pupa
*S. taigae*	A very short and irregular corridor, or a patch	Outer layer composed of two pieces of leaf tissue	No sclerotized zones on tergite of T2 and T3	1. Hind leg reaching middle of A6; 2. A single row of spines on A2–8.
*A. mesospilella*	A slender line, same in width (Lepiforum)	Outer layer composed of several pieces of leaf tissue [8]	Paired small sclerotization on lateral side of tergite of T2 and T3 (Lepiforum)	1. Hind leg reaching middle of A6 (Lepiforum); 2. Not examined.
*A. unifasciata*	Not available	Outer layer composed of one piece of leaf tissue [8]	Paired small sclerotization on lateral side of tergite of T2 [8]	1. Hind leg reaching posterior margin of A8; 2. A single row of spines on A3–8 [8].
*P. acerifoliella*	A broad corridor or a patch [12]	Outer layer composed of one piece of leaf tissue (Pohl et al. 2015: Figure 13) [12], but can also be of two pieces	Large sclerotization on T2 and T3	1. Hind leg beyond anterior margin of A8; 2. A single row of spines on A2–8 [11].

## Data Availability

Available barcode sequences are deposited in a public dataset, DS-INCUR, in BOLD.

## Abbreviations

SDNU	Zoological Collection, Shandong Normal University, Jinan 250014, China
A1–A8	Abdominal segments 1–8
T1–T3	Thoracic segments 1–3
